# Second primary cancer after female breast cancer: Familial risks and cause of death

**DOI:** 10.1002/cam4.1899

**Published:** 2018-11-26

**Authors:** Guoqiao Zheng, Akseli Hemminki, Asta Försti, Jan Sundquist, Kristina Sundquist, Kari Hemminki

**Affiliations:** ^1^ Division of Molecular Genetic Epidemiology German Cancer Research Center (DKFZ) Heidelberg Germany; ^2^ Faculty of Medicine, Cancer Gene Therapy Group University of Helsinki Helsinki Finland; ^3^ Helsinki University Hospital Comprehensive Cancer Center Helsinki Finland; ^4^ Center for Primary Health Care Research Lund University Malmö Sweden; ^5^ Department of Family Medicine and Community Health, Department of Population Health Science and Policy Icahn School of Medicine at Mount Sinai New York City New York; ^6^ Department of Functional Pathology, School of Medicine, Center for Community‐based Healthcare Research and Education (CoHRE) Shimane University Matsue Japan

**Keywords:** cause of death, cumulative incidence, familial cancer, second cancer, survival rate

## Abstract

**Background:**

With continuous increases in survival rates following breast cancer (BC) diagnosis, the challenge of multiple primary cancers has become an issue. The data on familial risk of SPCs after BC diagnosis and the related mortality in BC patients are scarce.

**Methods:**

A total of 87 752 female BC patients were followed for SPC diagnoses and records of death. Relative risks (RRs) of SPC in BC patients who had first‐degree relatives (parents or siblings) affected by the same cancer were compared to the patients without family history. Causes of death were compared between patients with and without SPC.

**Results:**

After a median follow‐up of 5 years, 14 952 BC patients developed SPCs, among which 10 280 (68.8%) had first‐degree relatives diagnosed with cancer. Familial risks were significant for 14 site‐specific SPCs, and the highest risk was for second ovarian cancer (RR = 6.28, 95%CI: 4.50‐8.75), compared to those without family history (1.49, 1.34‐1.65). In patients with SPC, SPC was the main cause of death, including diverse cancers and BC in approximately equal proportions.

**Conclusions:**

Family history contributed to the excess number of patients with SPCs, and SPC was the leading cause of death in patients with SPC. Taking family history at diagnosis of BC may provide warning signs with regard to possible subsequent SPCs and may offer possibilities for counseling, intervention and management.

## Abstract 2

Family history contributed to the excess number of BC patients with SPC. SPC was found the leading cause of death in BC patients with SPC.

## 1 INTRODUCTION

Survival rates following cancer diagnosis have improved and increased the likelihood for multiple primary cancers.^1^, ^2^ Breast cancer (BC) is common, and survival is generally good resulting in multiple primary cancers diagnosed in many patients. In the Swedish and German cancer registries, second primary cancers (SPCs) after BC account respectively for 11.8% and 10.9% of all SPCs.^3^ Known causes for multiple primary cancers in BC patients include radiotherapy, chemotherapy, hormonal and genetic factors.^1^, ^2^, ^4^ High risks for contralateral BC are well known and associate with familial BC although many such patients lack a family history of BC.^5^ Gene mutations in *BRCA1/2* constitute the dominant germline cause for heritable BC; these mutations are estimated to account for 1.4% of all BCs in European and African American populations.^6^ They also confer a high risk of ovarian cancer and a smaller but still increased risk of many other cancers.^7^ Although population‐level data on higher order multiple cancers (third, fourth etc primary cancers) should increase along with SPCs, they are often associated with genetic predisposition in the context of cancer syndromes.^1^, ^4^, ^8^, ^9^ In cancers featuring good survival, such as Hodgkin lymphoma and testicular cancer, SPCs are the main cause of death.^1^, ^10^, ^11^, ^12^, ^13^

Here we investigate risk for and mortality due to multiple primary cancers in BC patients. The resource used was the Swedish Family‐Cancer Database covering the Swedish population essentially through more than a century with linked cancers from the national cancer registry.

## 2 METHODS

Our Swedish Family‐Cancer Database was created by incorporating the Multigeneration Register, National Cancer Registry (started in 1958), national censuses and Cause of Death Register. This database includes all Swedish people born after 1931 (offspring generation) and their biological parents (parental generation). The latest version of the Swedish Family‐Cancer Database contained 16.1 million individuals among which 2.0 million were cancer patients recorded to the end of 2015. The follow‐up for cancer in the offspring generation was started from the beginning of 1958, the birth year, or the immigration year, whichever came latest. The follow‐up was terminated when a person was diagnosed with cancer, emigrated or died, or at the end of 2015, whichever came first. Follow‐up for SPC was started at diagnosis of first BC and terminated at diagnosis of SPC, emigration, death, or at the end of 2015.

Family history was defined through first‐degree relatives (parents or siblings). In situ cancers in breast, colorectum, melanoma and squamous cell (SCC) skin in family members were also included because familial risk in these in situ cancers are approximately equally as high as in invasive cancers.^14^ For the overall risk calculation, only family history of invasive cancers was considered. Cumulative incidence of SPC with or without a family history of concordant cancer was calculated considering death as a competing event. Poisson regression model was employed to estimate relative risks (RRs) and corresponding 95% confidence intervals (CIs) for SPC in the offspring generation by comparing the incidence of cancer as SPC in BC with or without family history to the incidence of the same cancer as a first primary cancer in general population of the database, which had neither a family history of SPC nor BC (except for risk estimation of second BC). Significance levels were indicated also for 1% and 0.1%. Trend test was performed to compare RRs of SPC estimated from Poisson regression in patients who had first‐degree relatives affected by the same cancer (positive family history) to the patients without family history (negative family history). Potential confounders, including age group (5‐year bands), calendar period (5‐year bands), residential area (large cities, South Sweden, North Sweden, or unspecified) and socioeconomic status (blue‐collar worker, white‐collar worker, farmer, private, professional, or other/unspecified) were added to the model as covariates. All the hypothesis tests were 2‐sided.

Underlying and contributing cause of death were obtained from the Swedish Cause of Death Register and were recorded according to the WHO International Classification of Diseases (ICD‐7, 1961; ICD‐8, 1969‐1986; ICD‐9, 1987‐1996; ICD‐10, 1997‐2015). All cancer related deaths were stratified into BC, SPC, higher order primary cancer and “other cancer”. In BC patients with second BC the cause of death was assigned to BC even though it was not known whether first or second BC killed the patient. Higher order primary cancer classification included patients who died of third, fourth or fifth primary cancer. “Other cancer” included cancers diagnosed at the issue of death certificates and they were not the first cancer, SPC or higher order primary cancer. These death certificate notifications are not counted by the Swedish Cancer Registry, opposite to the other Nordic Cancer Registries.^15^, ^16^, ^17^ Upon scrutiny of these cancers we found that they included often cancer of unknown primary (CUP). In our previous analyses these cancers have been interpreted as metastases.^18^, ^19^ If the death certificate notification matched the organ site of the reported primary cancer, it was classified as BC but in most cases such an assignment could not be made and the classification was to “other cancer”. Other non‐neoplastic causes of death were reported as “other cause”. Statistical analyses were done with SAS version 9.4.

The study was approved by the Ethical Committee of Lund University without requirement for informed consent, and the study was conducted in accordance with the approved guidelines.

## 3 RESULTS

A total of 87 752 BC patients were identified in women in the offspring generation born after 1931 (Table 1), for whom the RRs were estimated in Table 2. The number of BC patients in the parental generation was 166 083. The median age at diagnosis of BC was 55 years. A total of 14 952 patients (17.0%) were diagnosed with SPC at the median age of 63 years, and for 8653 (57.9%) of them, SPC was BC. Among 8626 patients whose information was available, 42.1% were second BC in the same breast and 57.9% were contralateral. The median follow‐up time from diagnosis of first BC was only 1 year to second BC whereas it was 8 years to other SPC. Among patients diagnosed with SPC, 68.8% had cancer family history. In familial SPC, 2228 (21.6%) SPCs were the same (concordant) cancer that was diagnosed in the family members, and for 8052 (77.4%) patients it was a discordant familial cancer. Among the patients with SPCs, 2543 (17.0%) were diagnosed with third primary cancers. A total of 18 998 (21.6%) death cases were observed in BC patients, 4828 of which had SPC diagnoses (fatality rate of 32.3% in BC with SPC). For patients with SPCs with and without family history the overall fatality rate was equal, 32.6% and 31.5%, respectively. However, the fatality rate for the patients without SPCs (16.1%) was half of that for the patients with SPCs.

**Table 1:** Demography of the breast cancer population followed during the period of 1958‐2015

No. of females followed	4 216 676
Number of cases	
A. No. of BC diagnoses	87 752
B. No. of SPC diagnoses in BC patients	14 952 (17.0% of all BC survivors, B/A)
C. Familial SPC	10 280 (68.8% of all BC survivors with SPC, C/B)
D. Familial SPC (concordant)	2228 (21.6% of all familial SPC, D/C)
E. Familial SPC (discordant)	8052 (77.4% of all familial SPC, D/C)
F. No. of third primary cancer diagnoses	2543 (17% of all BC survivors with SPC, F/B)
Number of deaths	
G. Deaths among all BC patients	18 998 (21.6% of all BC patients, G/A)
H. Deaths among BC patients with SPC	4828 (32.3% of all diagnosed with SPC, H/B)
I. Deaths among SPC patients with positive family history	3356 (32.6% of all familial SPC diagnoses, I/C)
J. Deaths among SPC patients with negative family history	1472 (31.5% of all SPC patients with negative family history, J/(B‐C))
K. Deaths among BC patients without SPC	14 170 (16.1% of all BC survivors without SPC, K/(A‐B))

BC, breast cancer; SPC, second primary cancer.

**Table 2:** Familial risk for second primary cancers among breast cancer patients

Site of second primary cancer	Breast cancer patients						*P*‐Trend
	Negative family history			Positive family history			
	N	RR	95%CI	N	RR	95%CI	
UAT	138	***1.38***	1.16‐1.64	5	2.31	0.96‐5.56	0.17
Esophagus	44	***1.56***	1.15‐2.11	0	‐	‐	‐
Stomach	93	***1.37***	1.11‐1.69	8	***3.14***	1.57‐6.28	**0.003**
Small intestine	46	***1.54***	1.14‐2.07	0	‐	‐	‐
Colorectum	683	***1.17***	1.08‐1.26	112^a^	***1.50***	1.25‐1.81	**<0.001**
Colon	499	***1.16***	1.06‐1.27	45	**1.35**	1.01‐1.81	**<0.001**
Rectum	233	**1.15**	1.01‐1.32	18	***1.90***	1.19‐3.01	**<0.001**
Anus	37	1.38	0.99‐1.92	0	‐	‐	‐
Liver	139	***1.25***	1.06‐1.49	7	**2.54**	1.21‐5.32	**0.007**
Pancreas	176	**1.20**	1.03‐1.39	9	***2.35***	1.22‐4.52	**0.02**
Nose	9	1.29	0.66‐2.51	0	‐	‐	‐
Lung	744	***1.54***	1.43‐1.66	96	***2.93***	2.40‐3.58	**<0.001**
Breast	6744	3.90	3.80‐4.00	1909^b^	***4.89***	4.67‐5.12	**<0.001**
Cervix	95	1.07	0.87‐1.31	2	1.58	0.39‐6.32	0.16
Endometrium	627	***1.42***	1.31‐1.54	37	***3.32***	2.40‐4.58	**<0.001**
Uterus	1	2.38	0.32‐17.49	0	‐	‐	‐
Ovary	364	***1.49***	1.34‐1.65	35	***6.28***	4.50‐8.75	**<0.001**
Other female genitals	53	1.09	0.83‐1.44	0	‐	‐	‐
Kidney	180	***1.44***	1.24‐1.68	7	**2.12**	1.01‐4.44	0.24
Bladder	189	***1.30***	1.12‐1.51	14	***2.21***	1.31‐3.74	**0.005**
Melanoma	423	***1.32***	1.19‐1.45	26	***1.97***	1.34‐2.89	**<0.001**
Skin (SCC)	339	***1.35***	1.21‐1.51	79^c^	***3.47***	2.78‐4.33	**<0.001**
Eye	25	1.42	0.95‐2.12	0			
Nervous system	204	1.01	0.88‐1.17	6	1.16	0.52‐2.57	0.10
Thyroid gland	90	***1.72***	1.39‐2.12	1	2.73	0.38‐19.42	0.31
Endocrine gland	172	**1.17**	1.01‐1.37	8	***3.50***	1.75‐6.99	**0.004**
Bone	10	1.83	0.96‐3.48	0	‐	‐	‐
Connective tissue	66	***2.15***	1.67‐2.76	0	‐	‐	‐
Non‐Hodgkin lymphoma	224	***1.21***	1.05‐1.38	10	**2.03**	1.09‐3.77	**0.01**
Hodgkin lymphoma	15	1.29	0.77‐2.17	0	‐	‐	‐
Myeloma	79	1.06	0.85‐1.33	2	2.08	0.52‐8.31	0.15
Leukemia	222	***1.38***	1.20‐1.57	5	1.32	0.55‐3.17	0.18
CUP	253	***1.37***	1.21‐1.55	10	1.82	0.98‐3.38	**0.01**
All cancers^d^	4672	***3.00***	2.91‐3.09	10280	***3.54***	3.46‐3.62	**<0.001**
All cancers^e^	1905	***1.25***	1.19‐1.31	4394	***1.53***	1.49‐1.58	**<0.001**

For specific cancer as SPC, the reference group is the general population in the database had neither a family history of SPC nor BC (except for risk estimation of second BC), which is used to calculate the incidence of this specific cancer as first primary cancer.

UAT, upper aerodigestive tract; SCC, squamous cell carcinoma; CUP, cancer of unknown primary.

Bolding, italic and underlining indicate that the 95%CI, 99%CI and 99.9%CI did not overlap with 1.00 respectively.

a 12 of the 112 cases had only family history of in situ colorectal cancer.

b 100 of the 1909 cases had only family history of in situ BC.

c 41 of the 79 cases had only family history of in situ skin cancer.

d Breast cancer is included into all cancers.

e Breast cancer is excluded from all cancers.

The impact of family history on the risk of SPCs was assessed in Table 2. An increased risk for SPC was found in BC patients, who had a first‐degree relative diagnosed with any cancer (RR, 3.54, 95%CI, 3.46‐3.62) compared to patients without family history (3.00, 2.91‐3.09). When only considering SPCs other than BC, the respective RRs were 1.53 (1.49‐1.58) and 1.25 (1.19‐1.31), translating to an attributable risk proportion of 18.3% ((1.53‐1.25) /1.53) for family history of cancer. For site‐specific cancer, the trend tests were significant for 14 SPCs (See Table 2). The highest risk for second malignancy was shown for ovarian cancer (6.28, 4.50‐8.75 with family history vs 1.49, 1.34‐1.65 without family history). For second BC, the RRs were very high in patients with or without family history (4.89 and 3.90, respectively). The largest numbers of familial SPCs were observed for BC (1909), followed by colorectal (112), lung (96) and skin (79) cancers.

**Figure 1:**
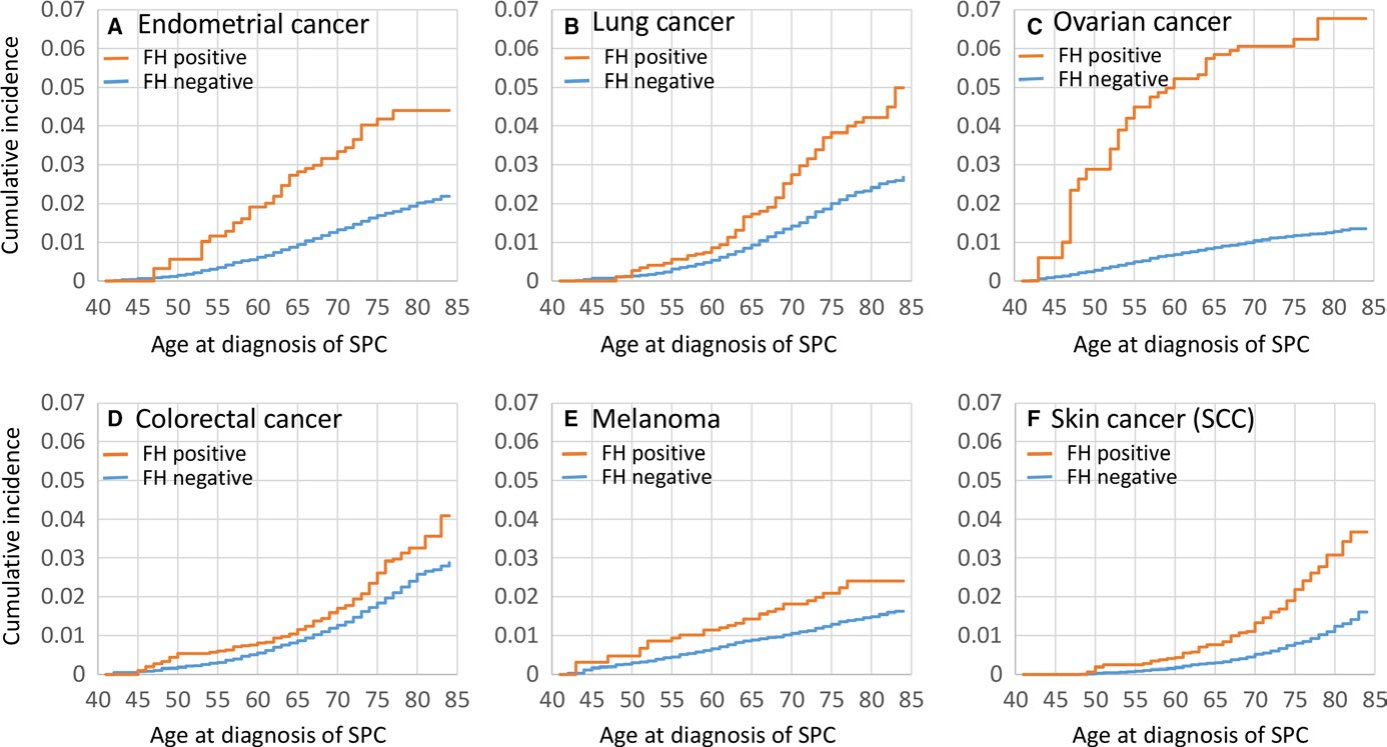
Cumulative incidence of second primary cancers (SPCs) according to family history and age at diagnosis of SPCs among breast cancer patients. Figures from A to F are respectively for endometrial, lung, ovarian and colorectal cancers, melanoma and skin cancers (SCC). SPC, second primary cancer. FH positive/negative, BC patients with/without a family history of specific cancer (for example, endometrial cancer in (A) in first‐degree relatives

Cumulative incidence rates for six cancer sites with most familial second primary cancer cases including endometrial, lung, ovarian, colorectal and skin cancers, and melanoma are shown in Figure 1A‐F according to age at diagnosis of SPCs. Shown are plots for SPCs with a family history of concordant cancer (orange curves) and without a family history (blue curves). For the cancers listed, the cumulative incidence with a family history was higher than that without family history at each age. A large difference between the cumulative incidence of SPCs with and without family history was observed for ovarian cancer; at the age of 75 years, the cumulative incidence with a family history reached 6%, in contrast to 1% when lacking a family history. The incidence rate of the corresponding second cancer at the specific age group is shown in Figure S1.

Table 3 shows the cause of death in BC patients with or without SPCs according to follow‐up time from diagnosis of BC. Among patients with SPCs, BC (mostly including second BC) and SPC were the dominant causes of death through the follow‐up periods. Deaths due to non‐BC SPCs accounted for 34.3% of all deaths, but adding BC patients with second BC (30.6%),combined SPC accounted for 64.9% of all deaths. The proportion of deaths due to second BC decreased from 42.6% in the first year of diagnosis of BC to 23.2% after 10 years of diagnosis. The proportion of patients, who died of non‐breast SPCs was stable at around 30%‐35%. In the first year after BC diagnosis, 2.1% patients died of higher order primary cancer and in the longest follow‐up time the proportion increased to 7.6%. For women diagnosed only with BC, the major cause of death was BC throughout the follow‐up time and other causes accounted for around 15% but increased to 32.1% of all death cases in the follow‐up time after 10 years.

**Table 3:** Cause of death in breast cancer patients with or without second primary cancer according to follow‐up time from breast cancer diagnosis

Breast cancer	Cause of death		<1 y (N, % in column)	1‐4 y (N, % in column)	5‐10 y (N, % in column)	>10 y (N, % in column)	All (N, % in column)
With SPC	Breast cancer	a	11 (7.7)	86 (10.1)	138 (9.2)	211 (9.1)	466 (9.6)
		b	61 (42.6)	344 (40.4)	533 (35.4)	540 (23.2)	1478 (30.6)
	SPC		40 (30.0)	258 (30.3)	543 (36.0)	815 (35.0)	1656 (34.3)
	Higher order primary cancer		3 (2.1)	26 (3.0)	56 (3.7)	177 (7.6)	262 (5.4)
	Other cancers		11 (7.7)	54 (6.3)	100 (6.6)	252 (10.8)	417 (8.6)
	Other causes		17 (11.9)	83 (9.8)	136 (9.0)	333 (14.3)	569 (11.8)
	All (N, % in row)		143 (3.0)	851 (17.6)	1506 (31.2)	2328 (48.2)	4828 (100.0)
Without SPC	Breast cancer		817 (77.0)	4032 (86.2)	4006 (81.1)	2190 (62.7)	11045 (77.9)
	Other cancers		48 (4.5)	104 (2.2)	152 (3.1)	184 (5.3)	488 (3.4)
	Other causes		196 (18.5)	541 (11.6)	779 (15.8)	1121 (32.1)	2637 (18.6)
	All (N, % in row)		1061 (7.5)	4677 (33.0)	4937 (34.8)	3495 (24.7)	14170 (100.0)

SPC, second primary cancer.

Higher order primary cancer, 3rd, 4th or 5th primary cancer.

a, Breast cancer patients diagnosed with non‐breast second primary cancer and dying of breast cancer; b, Breast cancer patients diagnosed with second breast cancer and dying of breast.

Table S1 shows the same data as Table 3 but covers only the follow‐up period from 2001 to 2015. Among patients with second BC diagnoses the overall proportions of deaths due to BC decreased from 30.6% in Table 3 to 28.1% in Table S1. A concomitant increase was observed in deaths due to non‐BC cancers (from 34.3% to 38.5%).

**Figure 2:**
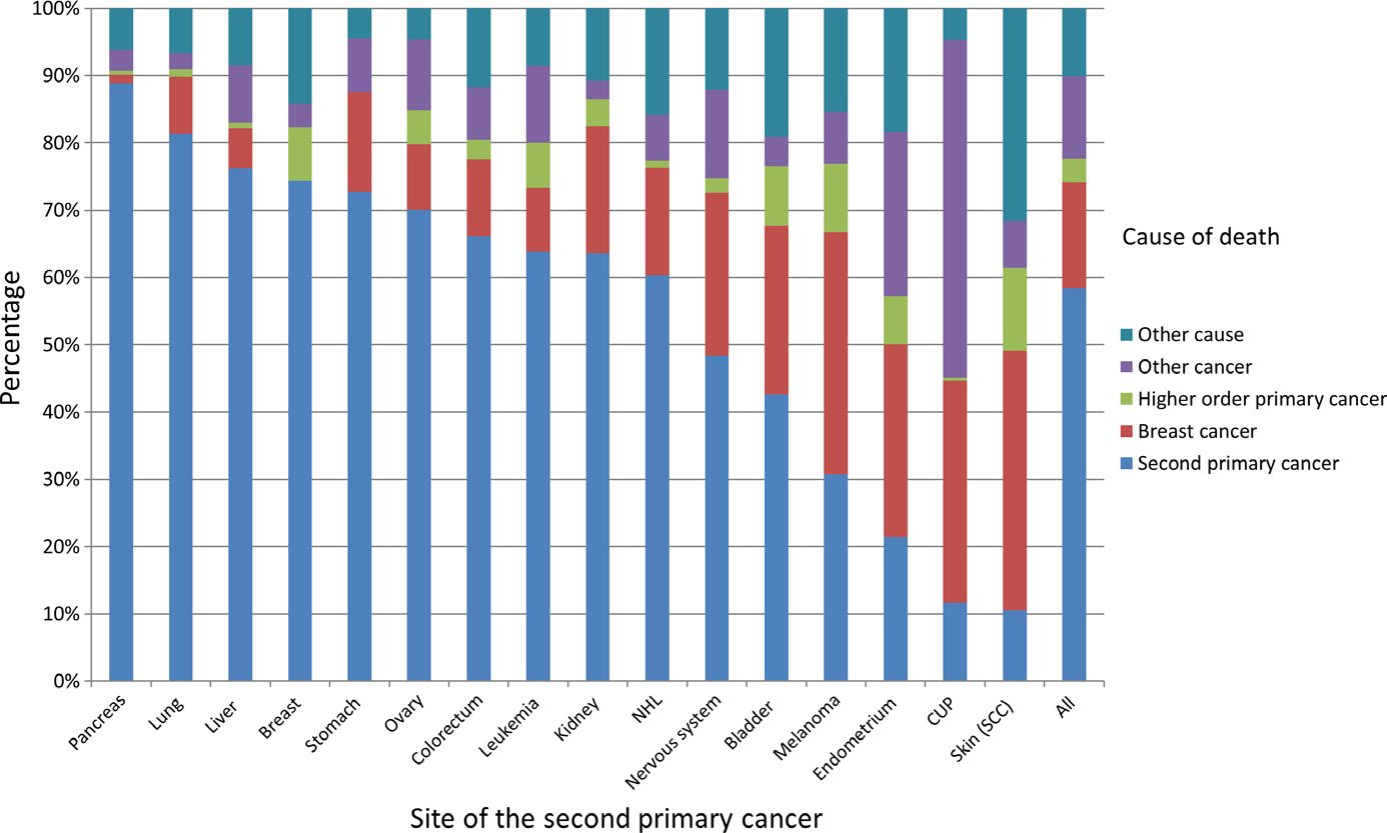
Distribution of cause of death: second primary cancer, breast cancer, higher order primary cancer, other cancers and other causes among breast cancer patients with second primary cancer. Only cancer sites with more than 50 death cases are displayed

Figure 2 displays the distribution of the cause of death in patients with SPCs when SPC conferred at least 50 deaths. Second pancreatic, lung, liver, breast, stomach and ovarian cancers each accounted for more than 70% of the deaths in patients with the corresponding SPC, while skin and endometrial cancers and CUP appeared least fatal. The relative contributions of deaths were reversed for deaths due to BC and other causes which were highest for the least fatal SPCs. Other cancers were a minor cause of death, except for CUP for which half of the deaths were due to other causes. Details of the distribution of the cause of deaths for all SPCs are shown in Table S2.

## 4 DISCUSSION

The proportion of BC patients diagnosed with SPCs was 17.0%, which interestingly exactly matched the proportion for third primary cancers among SPCs. Second BC was the main SPCs, accounting for more than half of the cases with SPCs. The median follow‐up time from diagnosis of first BC to second BC was much shorter than to the non‐breast SPCs and many BCs were diagnosed synchronously. In patients diagnosed with SPCs, the main cause of death was SPCs (64.9%), among which close to half of were second BCs. For the patients without SPCs, BC accounted for most deaths (77.9%).

Risk of SPCs in BC patients has been the subject of many studies, and second endometrial, ovarian, stomach and colon cancers, and melanoma were reported to occur most frequently according to a review.^20^ Apart from these cancers, increased risks of more SPCs were found in the current study, and importantly, family history contributed to the risk for the SPCs. Risk of second BC was very high irrespective of family history, indicating that most BC patients had a greater risk of developing second BC than other SPC. A family history of ovarian cancer in BC patients was associated with more than a 4 fold risk of second ovarian cancer, liver and endocrine gland cancers a 3 fold risk, skin (SCC) cancer a 2.5 fold risk, endometrial cancer more than a 2 fold risk, pancreatic and lung cancers close to a 2 fold risk cancer, and rectal, kidney, bladder cancers, melanoma and non‐Hodgkin lymphoma around a 1.5 fold risk. The increased risks for familial SPCs may be a consequence of complex genetic and environmental factors, including reproductive and other behavioral factors. Mutations in genes such as *BRCA1/2* are likely to contribute to the high familial risk of second breast and ovarian cancers,^21^, ^22^ as well as the risk of pancreatic cancer and melanoma.^23^ Hormonal level and reproductive factors may also contribute to the increased familial risk of breast, ovarian and endometrial cancers. Smoking is probably related to the increased familial risk of second lung, bladder and kidney cancers, and alcohol consumption to liver cancer.

The fatality rate in patients with SPC (32.3%) was twice as high as in patients without SPC (16.1%). This is possibly due to worse survival in most cancers other than BC, which was illustrated by high fatality rate in second stomach, pancreatic and liver cancers and CUP (Figure 2). For cancers with relatively good survival, such as melanoma, endometrial and skin cancers when diagnosed as SPC, the leading cause of death was BC. However, the fatality rate in patients with SPCs was not dependent on the family history of cancer, consistent with the literature reporting that family history does not impact fatality rate of BC patients.^24^, ^25^

It is important to consider some quality parameters in the context of the present data. SPCs undergo the same rigorous histological diagnostics as are applied for first cancers in the Swedish cancer registration system which is mandated to request separate and consistent tumor notifications from clinicians and pathologist. Accordingly an ad hoc study on the diagnostic accuracy of second neoplasms in the Swedish Cancer Registry found 98% to be correctly classified; no recorded SPC was found to be a metastasis.^26^ However, tumors in the same organs are more problematic as the distinction between a novel neoplasia and a recurrence is subject to tumor histology and anatomical location; in paired organs contralateral presentation, such as in BC, may favor calling of two separate tumors.^17^ Cause of death registration is done by the registrar using the relevant information regarding circumstances around the deaths. One study performed in Sweden, based on international standards, indicated 77% agreement between the cause of death from death certificates and the cause of death expected based on case summaries, and highest agreement was with malignant neoplasms as the underlying cause of death.^27^ A contributing factor to the accuracy of death certificates on cancer patients in Sweden is that 85% of patients die in hospitals and for more than 90% of cancer deaths the related hospital journals have been the base for issuing the death certificate.^28^, ^29^ Some clinical and behavioral factors are not available in the database, such as hormone receptor status, treatment for the BC, smoking and physical activity. However we adjusted socioeconomic status, which can reduce the confounding to some extent.

In conclusion, 17% of BC patients were diagnosed with SPCs in our database, among which 68.8% were familial. For BC patients with family members diagnosed with cancer, 18.3% of non‐breast SPCs were attributed to family history. High familial risks were found in cancers that share genetic, reproductive or behavioral factors with BC. SPC was the leading cause of death in the patients diagnosed with SPCs. Our results suggest that family history gives guidance on potential risk of SPCs and it may help counseling about risk factors and early detection of SPC in BC survivors. The impact of family history on SPCs was remarkably high which contributed to the overall high death rate by SPCs. Any attempts to increase survival in BC needs to counter the challenge of SPCs.

## CONFLICT OF INTEREST

AH is shareholder in Targovax ASA. AH is employee and shareholder in TILT Biotherapeutics Ltd. Other authors declared no conflict of interest.
