# Supplementary figures and images for: Second primary cancer after female breast cancer: Familial risks and cause of death
Source: Cancer Med. 2018 Nov 26;8(1):400–7. doi: 10.1002/cam4.1899 (PMC6346247; doi:10.1002/cam4.1899)

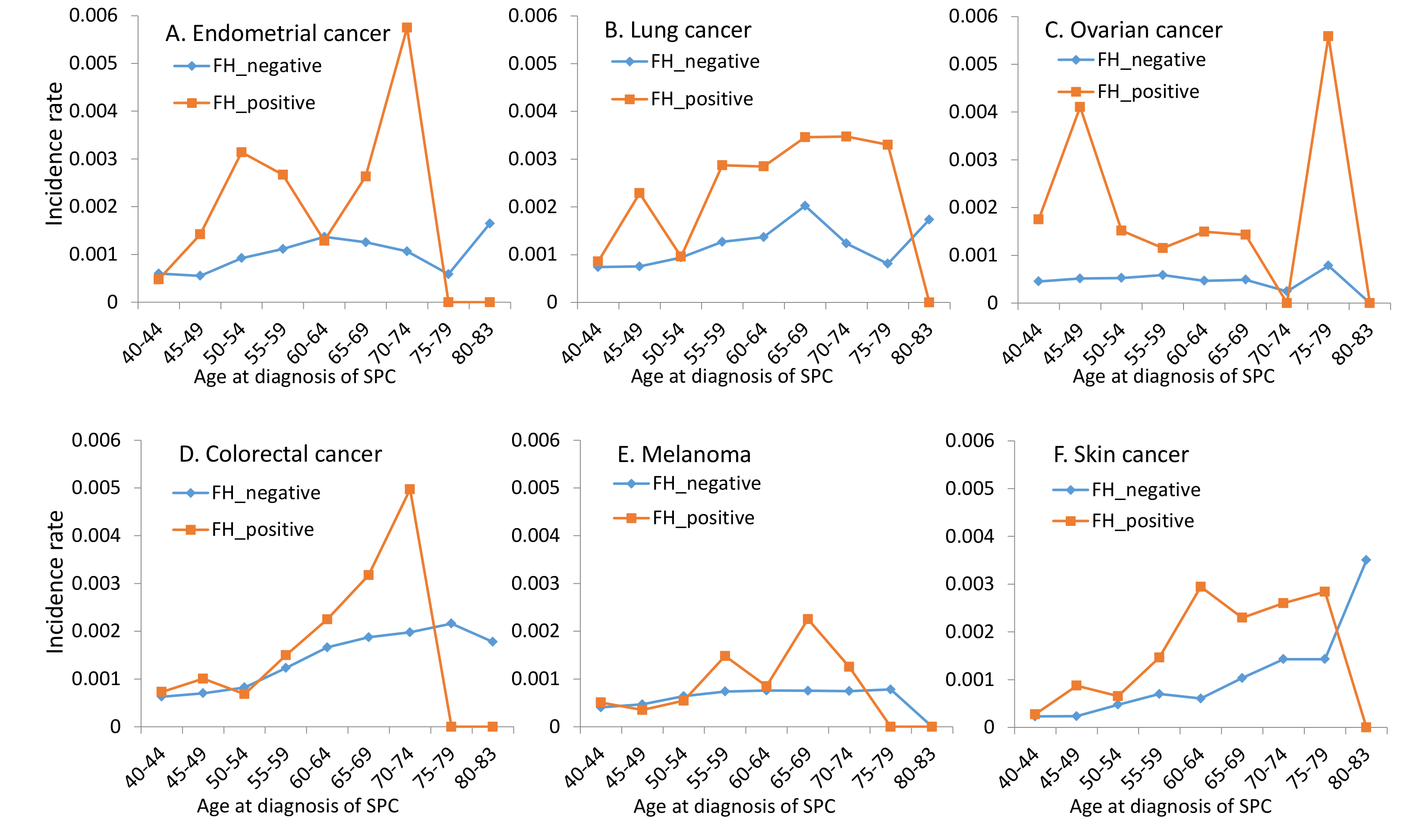

Supplement: Supplementary file 1 [file CAM4-8-400-s001.tif]
