# Supplementary material for: Second primary cancer after female breast cancer: Familial risks and cause of death
Source: Cancer Med. 2018 Nov 26;8(1):400–7. doi: 10.1002/cam4.1899 (PMC6346247; doi:10.1002/cam4.1899)
Supplement: Supplementary file 2 [file CAM4-8-400-s002.docx]

Supplementary table 1. Cause of death in breast cancer patients with or without second primary cancer according to follow-up time from breast cancer diagnosis (2001-2015)

| Breast cancer | Cause of death | | <1 year  (N, % in column) | 1-4 years  (N, % in column) | 5-10 years  (N, % in column) | >10 years  (N, % in column) | All  (N, % in column) |
| --- | --- | --- | --- | --- | --- | --- | --- |
| With SPC | Breast cancer | a | 8 (7.1) | 55 (9.4) | 70 (8.8) | 25 (10.7) | 158 (9.2) |
|  |  | b | 47 (41.6) | 194 (33.2) | 198 (25.0) | 46 (19.7) | 485 (28.1) |
|  | SPC | | 32 (28.3) | 203 (34.8) | 345 (43.5) | 83 (35.6) | 663 (38.5) |
|  | HPC | | 3 (2.6) | 25 (4.3) | 34 (4.3) | 17 (7.3) | 79 (4.6) |
|  | Other cancers | | 9 (8.0) | 36 (6.2) | 61 (7.7) | 26 (11.2) | 132 (7.7) |
|  | Other causes | | 14 (12.4) | 71 (12.2) | 85 (10.7) | 36 (15.4) | 206 (12.0) |
|  | All  (N, % in row) | | 113 (6.6) | 584 (33.9) | 793 (46.0) | 233 (13.5) | 1723 (100.0) |
|  | | | | | | | |
| Without SPC | Breast cancer | | 538 (74.0) | 2010 (81.1) | 1591 (72.6) | 268 (62.6) | 4407 (75.7) |
|  | Other cancers | | 36 (5.0) | 74 (3.0) | 91 (4.2) | 15 (3.5) | 216 (3.7) |
|  | Other causes | | 153 (21.0) | 393 (15.9) | 510 (23.3) | 145 (33.9) | 1201 (20.6) |
|  | All  (N, % in row) | | 727 (12.5) | 2477 (42.5) | 2192 (37.6) | 428(7.4) | 5824 (100.0) |

a, breast cancer patients diagnosed with non-breast second primary cancer and dying of breast cancer; b, breast cancer patients diagnosed with second breast cancer and dying of breast; SPC, second primary cancer; HPC, higher order (3^rd^, 4^th^ or 5^th^) primary cancer

Supplementary table 2. Causes of death in breast cancer patients diagnosed with second primary cancer

| Second cancer | Total number of  deaths and % of all patient with SPC | | Cause of death | | | | | | | | | |
| --- | --- | --- | --- | --- | --- | --- | --- | --- | --- | --- | --- | --- |
|  |  |  | Breast cancer | | SPC | | HPC | | Other cancers | | Other causes | |
|  | N | % | N | % | N | % | N | % | N | % | N | % |
| UAT | 46 | 32.2 | 11 | 23.9 | 17 | 40.0 | 5 | 10.9 | 7 | 15.2 | 6 | 13.0 |
| Esophagus | 33 | 75.0 | 1 | 3.0 | 28 | 84.8 | 1 | 3.0 | 3 | 9.1 | 0 | 0.0 |
| Stomach | 88 | 87.1 | 13 | 14.8 | 64 | 72.7 | 0 | 0.0 | 7 | 8.0 | 4 | 4.5 |
| Small intestine | 15 | 32.6 | 3 | 20.0 | 9 | 60.0 | 0 | 0.0 | 2 | 13.3 | 1 | 6.7 |
| Colorectum | 307 | 38.6 | 35 | 11.4 | 203 | 66.1 | 9 | 2.9 | 24 | 7.8 | 36 | 11.7 |
| Liver | 117 | 80.1 | 7 | 6.0 | 89 | 76.1 | 1 | 0.8 | 10 | 8.5 | 10 | 8.5 |
| Pancreas | 162 | 87.6 | 2 | 1.2 | 144 | 88.9 | 1 | 0.6 | 5 | 3.1 | 10 | 6.2 |
| Lung | 610 | 72.6 | 51 | 8.4 | 496 | 81.3 | 7 | 1.1 | 15 | 2.4 | 41 | 6.7 |
| Breast | 1990 | 23.0 | - | - | 1478 | 74.3 | 159 | 8.0 | 70 | 3.5 | 283 | 14.2 |
| Cervix | 47 | 48.4 | 10 | 21.3 | 24 | 51.1 | 3 | 6.4 | 5 | 10.6 | 5 | 10.6 |
| Endometrium | 168 | 25.3 | 48 | 28.6 | 36 | 21.4 | 12 | 7.1 | 41 | 24.4 | 31 | 18.4 |
| Ovary | 237 | 59.4 | 23 | 9.7 | 166 | 70.0 | 12 | 5.1 | 25 | 10.5 | 11 | 4.6 |
| Other female genitals | 22 | 41.5 | 1 | 4.5 | 6 | 27.3 | 5 | 22.7 | 6 | 27.3 | 4 | 18.2 |
| Kidney | 74 | 39.6 | 14 | 18.9 | 47 | 63.5 | 3 | 4.0 | 2 | 2.7 | 8 | 10.8 |
| Bladder | 68 | 33.5 | 17 | 25.0 | 29 | 42.6 | 6 | 8.8 | 3 | 4.4 | 13 | 19.1 |
| Melanoma | 78 | 17.4 | 28 | 35.9 | 24 | 30.8 | 8 | 10.2 | 6 | 7.7 | 12 | 15.4 |
| Skin (SCC) | 57 | 13.6 | 22 | 38.6 | 6 | 10.5 | 7 | 12.3 | 4 | 7.0 | 18 | 31.6 |
| Eye | 10 | 40.0 | 0 | 0.0 | 1 | 10.0 | 1 | 10.0 | 7 | 70.0 | 1 | 10.0 |
| Nervous system | 91 | 43.3 | 22 | 24.2 | 44 | 48.4 | 2 | 2.2 | 12 | 13.2 | 11 | 12.1 |
| Thyroid gland | 21 | 23.1 | 7 | 33.3 | 9 | 42.8 | 2 | 9.5 | 0 | 0.0 | 3 | 3.3 |
| Endocrine gland | 37 | 20.6 | 13 | 35.1 | 5 | 13.5 | 5 | 13.5 | 5 | 13.5 | 9 | 24.3 |
| Bone | 7 | 70.0 | 5 | 71.4 | 0 | 0.0 | 0 | 0.0 | 2 | 28.6 | 0 | 0.0 |
| Connective tissue | 32 | 48.5 | 7 | 21.9 | 11 | 34.4 | 2 | 6.2 | 7 | 21.9 | 5 | 15.6 |
| Non-Hodgkin lymphoma | 89 | 38.0 | 14 | 15.7 | 53 | 59.5 | 1 | 1.1 | 6 | 6.7 | 15 | 15.6 |
| Hodgkin lymphoma | 5 | 33.3 | 0 | 0.0 | 1 | 20.0 | 1 | 20.0 | 1 | 20.0 | 2 | 40.0 |
| Meyloma | 39 | 48.1 | 2 | 5.1 | 30 | 76.9 | 1 | 2.6 | 2 | 5.1 | 4 | 10.2 |
| Leukemia | 105 | 46.2 | 10 | 9.5 | 67 | 63.8 | 7 | 6.7 | 12 | 11.4 | 9 | 8.6 |
| CUP | 213 | 81.0 | 70 | 32.9 | 25 | 11.7 | 1 | 0.5 | 107 | 50.2 | 10 | 4.7 |
| All ^a^ | 2838 | 45.0 | 446 | 15.7 | 1656 | 58.4 | 103 | 3.6 | 347 | 12.2 | 286 | 10.1 |

a:second breast cancer was excluded from all cancers

SPC, second primary cancer; HPC, higher (3^rd^, 4^th^ or 5^th^) primary cancer; UAT, upper aerodigestive tract; SCC, squamous cell carcinoma; CUP, cancer of unknown primary.
